# Formulation of Caffeine–Hydroxypropyl-β-Cyclodextrin Complex in Hydrogel for Skin Treatment

**DOI:** 10.3390/gels11050326

**Published:** 2025-04-27

**Authors:** Lyubomira Radeva, Eleftheria Kalampalika, Yordan Yordanov, Petar D. Petrov, Virginia Tzankova, Krassimira Yoncheva

**Affiliations:** 1 Faculty of Pharmacy, Medical University of Sofia, 1000 Sofia, Bulgariayyordanov@pharmfac.mu-sofia.bg (Y.Y.); vtzankova@pharmfac.mu-sofia.bg (V.T.); 2 Institute of Polymers, Bulgarian Academy of Sciences, 1113 Sofia, Bulgaria; ppetrov@polymer.bas.bg

**Keywords:** caffeine, hydroxypropyl-β-cyclodextrin, complex, hydrogel, antioxidant activity

## Abstract

Caffeine is a well-known xanthine that possesses antioxidant effects that could contribute to its application in different skin disorders. In order to enhance its effects, approaches for improving its permeation and penetration through skin layers could be applied. This study emphasizes the preparation of caffeine–cyclodextrin complex and its formulation in carbopol hydrogel. The complex was developed at a 1:1 molar ratio between caffeine and hydroxypropyl-β-cyclodextrin. It was found that the complex enhanced the radical scavenging activity of caffeine against ABTS radical as well as the protective effects against H_2_O_2_-induced oxidative stress in L929 fibroblasts. Then, the complex was formulated in hydrogel by applying 1% carbopol. The spreadability and penetration of the loaded hydrogel were improved in comparison with the empty hydrogel. The results revealed that the system could be appropriate for therapies of skin disorders, and its wound healing abilities could be further investigated.

## 1. Introduction

Climate change associated with global warming and ozone depletion is expanding skin exposure to ultraviolet (UV) light, due to the passage of more UVB rays. This increases the risk of skin aging, which is related to dryness, deep wrinkles, loss of elasticity, and pigmentation. The risk of developing skin cancer has also significantly increased. According to literature data, skin cancer cases in the UK are expected to increase by 5000 per year in the middle of the 21st century [1]. In addition, changes in temperature, humidity, UV radiation, and air pollution negatively affect the skin microbiome. This would also have an impact on the epidemiology and severity of skin diseases such as atopic dermatitis, psoriasis, acne, and cancer [2]. One of the mechanisms of action of UV radiation is the induction of oxidative stress through the generation of reactive oxygen species (ROS) [3]. It is also known that the excessive production of ROS can hinder cutaneous wound healing or injured tissue regeneration since it could trigger processes such as necrosis, inflammation, and fibrotic scarring [4]. Therefore, the development of new strategies for overcoming the problems related to skin damage is gaining more interest.

Topical application of antioxidants can prevent problems related to the oxidative stress of skin cells. Caffeine (1,3,7-trimethylpurine-2,6-dione) is a natural antioxidant found in the fruits and leaves of plants such as coffee (*Coffea* spp.), tea (*Camelia sinensis*), cocoa (*Theobroma cacao*), cola (*Cola acuminate*), guarana (*Paullinia cupana*), yerba mate (*Ilex paraguariensis*), and others. The antioxidant effect of caffeine is mainly related to the scavenging of the hydroxyl radical (•OH). In addition, the further reaction of caffeine with this radical leads to the formation of products that have antioxidant activity themselves [5]. This effect of caffeine may favorably affect skin health by protecting it from UV-induced oxidative stress and sun damage [6]. The skin protection is additionally supported due to the capacity of caffeine to increase the expression of antioxidant enzymes such as superoxide dismutase (SOD) and catalase [7]. It has been reported that in concentrations in the range of 1–5%, caffeine ensures effective protection against oxidative damage [8]. Furthermore, caffeine promotes microcirculation and enhances the delivery of nutrients to skin cells, which can improve skin tone and texture. These effects of caffeine were implemented in the treatment of cellulite [9]. The anticellulite effect is enhanced by the lipolytic properties of caffeine that are related to (1) increase in intracellular concentrations of cyclic adenosine monophosphate by inhibiting phosphodiesterase enzymes in the adipose tissue; (2) activation of hormone-sensitive lipase; and (3) blockage of adrenergic receptors, preventing an excessive accumulation of fats [10,11].

However, the topical application of caffeine is limited because of its hydrophilicity. In particular, the skin permeability of caffeine is hindered due to the lipophilic nature of stratum corneum and hydrophilic properties of caffeine (log P of 0.07). Therefore, strategies for overcoming this issue are needed. For instance, the pretreatment with permeability enhancers resulted in enhanced permeation of caffeine through the skin [12]. It was also found that the incorporation of caffeine into nanoscale vesicles could increase its permeation across the stratum corneum as well as its penetration through the skin [13].

Cyclodextrins are widely used excipients for preparation of inclusion complexes of drugs aiming to improve their biopharmaceutical properties. They have been used in formulations for treatment of psoriasis, dermatitis, wounds, onychomycosis, and skin cancer, as well as in anti-aging and sunscreen products [14]. Moreover, cyclodextrins could improve bioavailability of drugs via modulation of their cutaneous permeation after the inclusion in the complexes [14]. For instance, ketoconazole-microemulsion-hydroxy-β-cyclodextrin system was obtained by Che et al. [15]. It was found that the inclusion into the cyclodextrin complex increased the skin retention and chemical stability of the drug. The storage stability and skin penetration of *Celastrus paniculatus* seed oil were also enhanced after complexation with hydroxypropyl-β-cyclodextrin [16]. Felton et al. discovered that the increase in the concentration of hydroxypropyl-β-cyclodextrin from 0 to 10% in inclusion complexes with oxybenzone led to increased transdermal permeation and skin accumulation [17]. Such inclusion complexes have been further incorporated into semisolid dosage forms for topical application. Bianci et al. incorporated the coumestrol-hydroxypropyl-β-cyclodextrin complex into hydroxypropyl methylcellulose hydrogel for wound healing [18]. A similar complex with minoxidil was also incorporated into alginate hydrogel for treatment of alopecia [19]. An inclusion complex between hydroxypropyl-β-cyclodextrin and thymol was included into carbopol hydrogel intending topical administration [20]. Such an incorporation into hydrogels is significantly appropriate since these forms could provide a humid environment and easy transmission of water vapors and oxygen, prevent invasion of microorganisms, and remove soluble toxins [21,22]. The formulation of cyclodextrin complex of caffeine in hydrogel could be an advantageous strategy for caffeine permeation, having in consideration a recent study that reported an enhanced skin permeation of caffeine via hydration of the stratum corneum [23].

The purpose of this research was the formation of a complex between caffeine and hydroxypropyl-β-cyclodextrin and its further incorporation into a carbopol hydrogel. The radical scavenging capacity of the caffeine complex was examined in the presence of ABTS and DPPH radicals. In addition, the protective effect of the complex against H_2_O_2_-induced oxidative stress in fibroblasts was also assessed. Furthermore, spreadability and rheology of the hydrogel containing caffeine complex were determined, aiming to prove its appropriateness for topical application.

## 2. Results and Discussion

### 2.1. Solubility Studies

**Figure 1:**
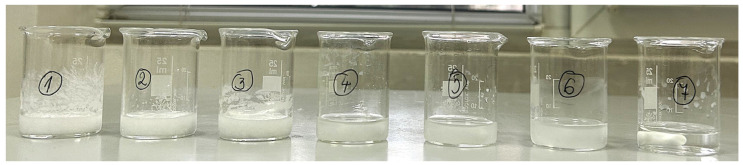
Images of pure caffeine (1) and complexes (2, 3, 4, 5, 6 and 7) prepared at a ratio 1:1, 1:2, 1:3, 1:4, 1:5 and 1:6 between caffeine and hydroxypropyl-β-cyclodextrin, respectively.

**Figure 2:**
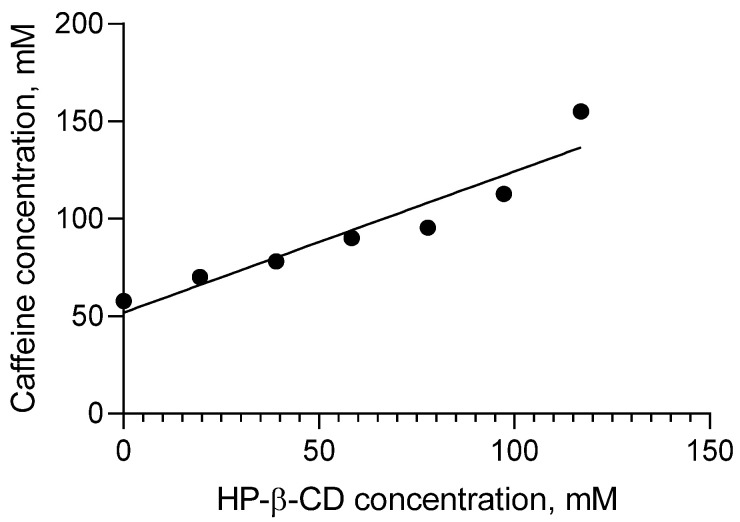
Phase-solubility profile of caffeine incorporated in the hydroxypropyl-β-cyclodextrin complex.

The complexes between caffeine and hydroxypropyl-β-cyclodextrin were prepared at different ratios (Figure 1). Phase solubility studies were performed aiming to examine the optimal ratio between hydroxypropyl-β-cyclodextrin and caffeine in the complex. Figure 2 shows that there is an Ap (positive-type isotherm) curve, which indicates the formation of soluble inclusion complexes and a higher-order complex with respect to cyclodextrin, meaning the presence of more than one molecule of cyclodextrin forming the complexes [24,25]. This could indicate a ratio 1:2 of drug to cyclodextrin in the complex [25]. The stability constant (K_1:1_) found in our study was 37.44 M^−1^ (Table 1), which could be considered as a suggestion of weak binding [24]. Terekhova et al. observed similar low values of stability constants for xanthine–hydroxyl-β-cyclodextrin (10.07 kg mol^−1^), caffeine–α-cyclodextrin (31 kg mol^−1^), and caffeine–β-cyclodextrin (30 kg mol^−1^) complexes [26,27]. However, according to Loftsson et al., this stability constant is significantly dependent on the intrinsic solubility [28]. Therefore, the high intrinsic solubility (S_0_) of caffeine, namely, 11.2 mg/mL, could be the reason for the lower value of the binding constant. Similar values of K_1:1_ were found for methamizole (S_0_ = 0.7 mg/mL) [28]. Therefore, we also calculated the complexation efficiency, which is less dependent on this intrinsic solubility. Moreover, it is important to determine this factor since it provides information about the solubilizing efficacy of the cyclodextrin [28,29]. The value of the complexation efficiency (CE) that we found was 2.16, which means that two from three cyclodextrin molecules are involved in the formation of complexes [28,29,30]. It is considered that the smaller amount of cyclodextrin is more advantageous [29]. A complex between caffeine and hydroxypropyl-β-cyclodextrin at a molar ratio of 1:1, which showed excellent stability and no signs of physical instabilities, was chosen for all the next experiments.

### 2.2. DPPH and ABTS Assays

**Figure 3:**
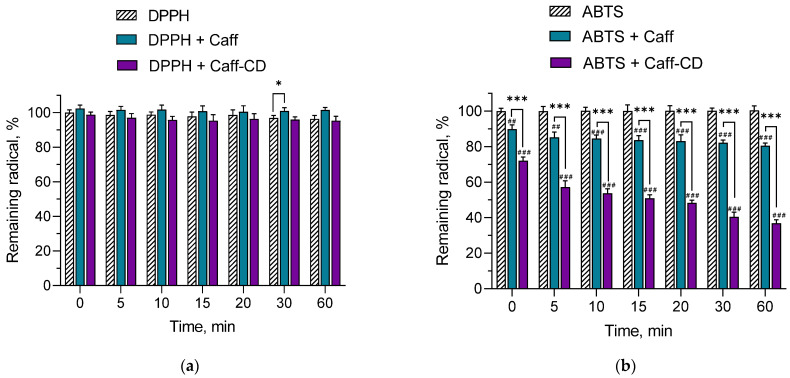
Radical scavenging activity of pure caffeine (Caff) and caffeine incorporated into the complex (Caff-CD) against DPPH (**a**) and ABTS (**b**) radicals. ## *p* < 0.01 and ### *p* < 0.001 vs. DPPH and ABTS groups; * *p* < 0.05 and *** *p* < 0.001 between pure caffeine and caffeine incorporated into the complex.

The radical scavenging activity of caffeine before and after the inclusion in the complex was examined via performing two non-cellular in vitro methods, namely, DPPH and ABTS assays (Figure 3). The scavenging ability of many compounds with antioxidant properties, such as lycopene [31], ellagic acid [32], and rosmarinic acid [33], has been enhanced after the formulation of cyclodextrin complexes. Our results revealed that neither the pure caffeine nor the caffeine incorporated into the complex showed scavenging activity against DPPH radicals (Figure 3a). However, the ABTS test showed that both samples possessed statistically significant scavenging activity against ABTS radical (Figure 3b). More importantly, the radical scavenging ability of caffeine was significantly enhanced after its inclusion in the cyclodextrin complex. Immediately after the mixing of the radical with the samples, the scavenging of ABTS approximated 10% for pure caffeine and 28% for the complex. Moreover, after 60 min of incubation, caffeine reduced the amount of the free radical to approximately 80%, while the complex reduced its concentration to 37%. The different effects of the complex against ABTS and DPPH radicals could be explained with the different solubilities of the radicals. DPPH assay is more suitable for hydrophobic antioxidants in comparison with ABTS, which can be used for both hydrophilic and lipophilic substances without being affected by ionic strength [34]. The steric accessibility is also a vastly important factor for the DPPH assay [34,35]. In the DPPH assay, the initial electron transfer is slower, which is explained with the sterically hindered DPPH radical site, which can be difficult to access. In addition, the ABTS assay is characterized by higher reactivity, which results in increased sensitivity [35]. Similar results were achieved by Chen et al., who reported that an inclusion complex between curcumin and β-cyclodextrin showed significantly better radical scavenging activity against ABTS radicals in comparison with DPPH radicals [36].

### 2.3. In Vitro Protective Effects Against H_2_O_2_-Induced Oxidative Stress

**Figure 4:**
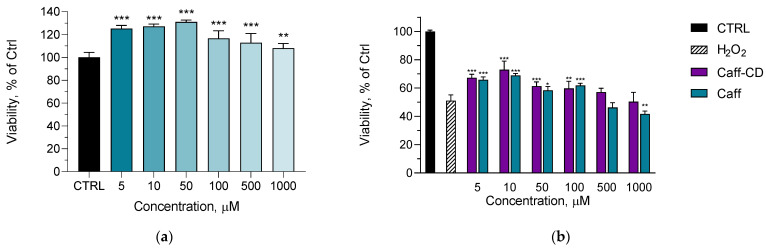
Cytotoxic effects of caffeine on L929 fibroblasts; ** *p* < 0.01 and *** *p* < 0.001 vs. control (**a**) and protective effects of pure caffeine (Caff) and caffeine incorporated into the complex (Caff-CD) against H_2_O_2_-induced oxidative stress in L929 fibroblasts; * *p* < 0.05, ** *p* < 0.01, and *** *p* < 0.001 vs. H_2_O_2_-treated control (**b**).

Considering the stronger antioxidant effect of the complex against ABTS radical, the next study aimed to examine the capacity of the complex to protect fibroblast cells against H_2_O_2_-induced oxidative stress. First, the in vitro cytotoxicity studies revealed that caffeine did not decrease the viability of L929 fibroblasts at the 5–1000 µM concentration range (Figure 4a). Moreover, it promoted the proliferation of the cells, especially in the lower concentrations (5, 10, and 50 µM). This could be explained with the ability of caffeine to facilitate the accumulation of cyclic adenosine monophosphate (cAMP) in cells, resulting in enhanced cell proliferation [37]. This observation corresponds to a previous study on orbital fibroblasts [38].

Further, the model of oxidative stress was performed by pretreatment of the cells with concentrations in the range established by the cytotoxic studies. Figure 4b presents the viability of the fibroblasts after pretreatment with pure caffeine (Caff) or the complex of caffeine and cyclodextrin (Caff-CD) and consequent induction of oxidative stress via H_2_O_2_ treatment. As shown, significant protection was registered when the cells were pretreated with pure caffeine and its complex form at 5, 10, 50, and 100 µM concentrations. At the highest concentrations, the pure caffeine potentiated the effect of H_2_O_2_, which was significant at 1000 µM concentration. In comparison, such an effect was not observed when the cells were pretreated with the complex. Therefore, there was a tendency for improvement of the protective effect when caffeine was in the complex. Similarly, Tiwari et al. observed that caffeine at 50 µM concentration showed protective effect against hyperoxia-induced damage on A549 human lung epithelial cells and MLE 12 SV40 transformed mouse epithelial cells, while a concentration of 1000 µM accelerated the toxic effect [39].

### 2.4. Incorporation of Caffeine Complex into Carbopol Hydrogel and Characterization of the Formulation

Hydrogels are widespread semisolid dosage forms for the treatment of a large number of skin complications. The application of hydrogels as protective formulations (e.g., sun protection) or for wound treatment is vastly appropriate since they possess the ability to provide a moist environment [40]. Carbopol, a cross-linked poly(acrylic acid), is one of the most common carriers for hydrogels. It is a non-toxic, non-irritating, and easy accessible gelling agent. The safety of carbopol hydrogel was proved in vitro and in vivo [41]. The study discovered that it had no cytotoxic effects on healthy keratinocytes (HaCaT cells) and murine epidermal cells (JB6 Cl 41-5a) and even enhanced cell proliferation. The hydrogel also showed good biocompatibility after application on skin of BALB/c nude mice as well as lack of toxicity after exposure to hen’s chicken embryo.

**Figure 5:**
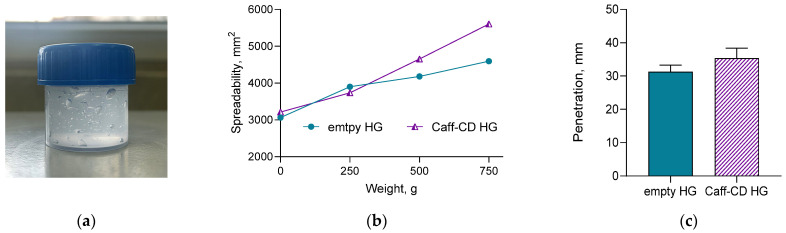
Image of the Caff-CD hydrogel (**a**), spreadability (**b**), and penetrometry (**c**) of the empty hydrogel (empty HG) and the hydrogel loaded with caffeine complex (Caff-CD HG).

The spreadability of semisolid dosage forms is a significantly important factor since the easy application, the skin area covered, and the effects depend on it. In our study, the spreadability of both the empty (empty HG) and the complex loaded with caffeine (Caff-CD HG) (Figure 5a) hydrogels were enhanced by increasing the weight, which indicates comfortable topical application and ensures the discussed advantages of hydrogels (Figure 5b). Similar values for carbopol hydrogel were reported by another study group too [41,42]. Moreover, the spreadability of the loaded hydrogel was higher than that of the empty one, which confirmed the observation of Zakzak et al. [41]. The spreadability factor of the hydrogel loaded with the complex was also higher in comparison with that of the empty hydrogel, namely, 10.57 vs. 10.04. Similarly, an increase in the penetration was registered for the loaded hydrogel. In particular, it was 35.4 mm vs. 31.3 mm for the empty one, which is another indication for enhanced application and good coverage of skin area (Figure 5c).

**Figure 6:**
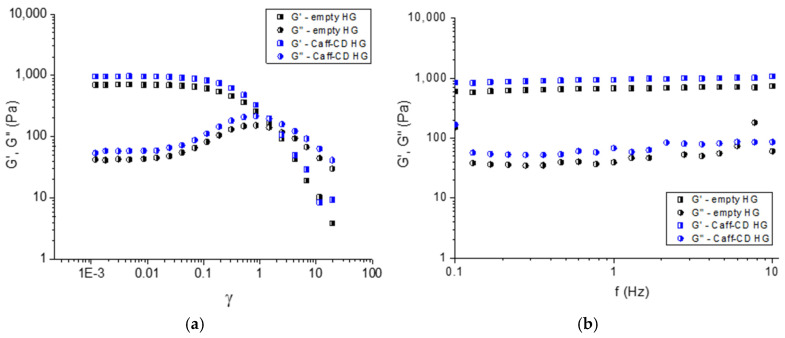
Variation in elastic (G′) and loss (G′′) moduli as a function of shear strain (**a**) and frequency (**b**).

Amplitude sweep tests were carried out to compare the behavior of empty (empty HG) and Caff-CD-loaded carbopol (Caff-CD HG) hydrogels regarding their structural stability with increasing the shear strain (γ) (Figure 6a). In particular, the study was focused on determining the gel to fluid transition. Generally, for an amplitude scan, the deflection of the measurement system was increased stepwise from γ = 0.001 to 20, while maintaining the frequency at a constant value (1 Hz). The linear viscoelastic region, observed on the left side of the plot at low values of the shear strain (γ = 0.001–0.02), indicated the behavior typical for hydrogel materials. In this region, the elastic (G′) and loss (G″) moduli were independent of the shear strain, and the elastic component dominated over the viscous component (G′ > G″). Increasing the shear strain caused gradual destruction of the polymer network and transition to a liquid phase. The initial raise of G″ (γ = 0.02–1) is related to the loss of more deformation energy as a result of internal friction during shearing. At a certain point, the applied stress overcame the internal resistance of the gel, and the polymer network tended to break down. Finally, the viscous component became dominant (G″ > G′), and the material began to flow. It seems that the incorporation of the Caff-CD complex into the hydrogel matrix did not affect the flow point (G′ = G″) of material, which was determined at γ = 1. On the other hand, G′ of the Caff-CD containing hydrogel was larger than G′ of the empty gel, indicating a slight reinforcing effect. Most probably this was due to the incorporation of rigid CD cyclic molecules. Next, oscillatory frequency scans were performed to describe the time-dependent behavior of the samples at a nondestructive deformation. In the frequency measurement (Figure 6b), the frequency of oscillation was increased in steps from 0.1 to 10 Hz, while the amplitude was kept constant (γ = 0.002). The two moduli were frequency-independent and G′ > G″ at the simulated conditions, ranging from slow motion on long time scales (low frequencies) to fast motion on short time scales (high frequencies). The results from oscillatory frequency tests confirmed that the studied materials behave as physical hydrogels at a low deformation.

### 2.5. In Vitro Release Studies

**Figure 7:**
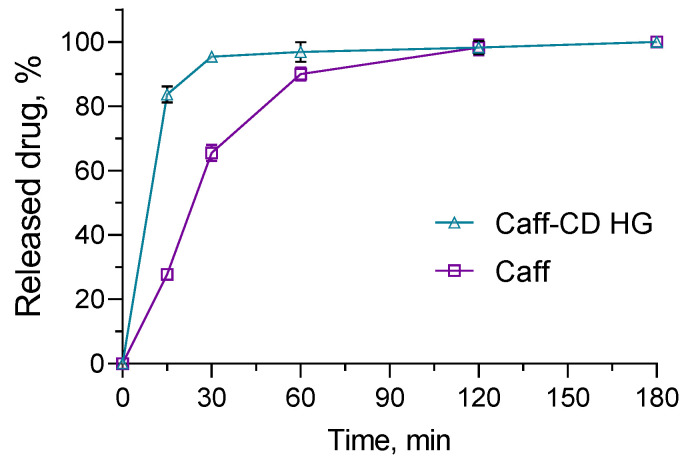
In vitro dissolution profiles of caffeine from carbopol hydrogel loaded with caffeine complex (Caff-CD-HG) and pure caffeine (Caff) at pH = 5.0.

The in vitro release tests were conducted in a buffer medium (pH 5.0) that represents the pH of healthy skin [43]. We observed an immediate release of the caffeine from the complex (less than 10 min). For comparison, the caffeine release from the hydrogel containing the complex of caffeine (Caff-CD HG) was slower. In particular, approximately 80% of the caffeine was released for the first 15 min, and the process continued for 180 min (Figure 7). In contrast, the slowest release was found for the pure caffeine (Caff). Namely, less than 30% was dissolved for 15 min, and 100% was reached after 120 min. Therefore, the formulation of the caffeine–cyclodextrin complex in the hydrogel led to faster dissolution, which would provide available caffeine for skin penetration. Similar results were observed by Ellah et al., who prepared carbopol 934 hydrogel loaded with spidroin [44]. Such a pattern could provide a high dose of caffeine delivered onto the skin, where it will be able to exert its topical pharmacological effects. Despite the fast release of caffeine from the system, the bioadhesive properties of the carpobol hydrogel could ensure long retention time, leading to improved penetration and pharmacological effects [45]. In addition, carbopol gel is reported to represent an appropriate vehicle for caffeine. Veryser et al. found that the diffusion coefficient and the partitioning of caffeine from carbopol gel was higher compared to Pluronic-based gel [46].

## 3. Conclusions

The development of an inclusion complex between caffeine and hydroxypropyl-β-cyclodextrin and its further incorporation in carbopol hydrogel could be considered a successful strategy for enhancing the skin application of caffeine. It was found out that two of three molecules of the cyclodextrin participated in the formation of the complex. Moreover, the complex was found to exert more pronounced radical scavenging activity against ABTS radical as well as to protect fibroblast cells against H_2_O_2_-induced oxidative stress stronger than pure caffeine solution. The developed carbopol hydrogel showed appropriate characteristics for skin application, namely, enhanced spreadability and penetration. Therefore, the formulation could be further examined for topical formulation intended for antioxidant therapy.

## 4. Materials and Methods

### 4.1. Materials

Caffeine, hydroxypropyl-β-cyclodextrin, carbopol 940, Dulbecco’s modified Eagle’s medium, fetal bovine serum (FBS), and L-glutamine were purchased from Sigma Chemical Co. (Merck, Darmstadt, Germany). The murine fibroblast L929 cell line was obtained from the European Collection of Cell Cultures (ECACC, Salisbury, UK).

### 4.2. Preparation of Caffeine–Hydroxypropyl-β-Cyclodextrin Complex and Solubility Studies

The complex between caffeine and hydroxypropyl-β-cyclodextrin was obtained via the incubation method. The solubility studies were performed according to previously described studies [30,47]. Different molar ratios between caffeine and the cyclodextrin were applied. First, hydroxypropyl-β-cyclodextrin was dissolved in 5 mL distilled water. Then, the determined amount of caffeine was added, and the system was stirred for 24 h at 700 rpm. Thereafter, the samples were centrifuged at 5500 rpm for 10 min (MPW-260, MPW Med. Instruments, Warsaw, Poland), and the concentration of caffeine in the supernatant was measured spectrophotometrically at 275 nm (Thermo Fisher Scientific, Waltham, MA, USA). The stability constant (*K*_1:1_) and complexation efficiency (CE) were calculated according to the following equations:

(1) $$K_{1\text{:}1}=\frac{Slope}{S_{0}(1-Slope)},$$

where *S*_0_ is the intrinsic solubility of caffeine (when no cyclodextrin is added).

(2) $$CE=\frac{Slope}{1-Slope}=\frac{D/CD}{CD}$$

where *D*/*CD* is the concentration of the complex between the cyclodextrin and the drug and CD is the concentration of the non-reacted cyclodextrin.

### 4.3. DPPH and ABTS Assays

The radical scavenging activity of caffeine and the caffeine complex was examined via DPPH [48] and ABTS [49,50] assays. For the DPPH assay, 1 mL solution of the caffeine complex or hydroalcoholic solution of the pure caffeine (30 mg/mL) was mixed with 1 mL of DPPH ethanol solution (440 µg/mL). Immediately after that, the absorbance of the reduced form of the radical was measured spectrophotometrically at 517 nm (Thermo Fisher Scientific, Waltham, MA, USA).

For the ABTS assay, aqueous solutions of ABTS and potassium persulfate were mixed, and the mixture was incubated in the dark at 25 °C for 16 h in order to activate the radical. After that, 1 mL of the caffeine complex or the hydroalcoholic solution of caffeine at corresponding concentrations was mixed with 1 mL 5% alcoholic solution of ABTS. The absorbance of the reduced radical was measured spectrophotometrically (Thermo Fisher Scientific, Waltham, MA, USA) at 734 nm.

### 4.4. In Vitro Studies on Cells

Cell viability studies and the H_2_O_2_-induced model of oxidative stress were performed on mouse fibroblasts L929 by applying neutral red assay [51]. The cells were seeded in 96-well plates at a cell density of 2 × 10^4^ and incubated overnight at standard conditions (37 °C, 5% CO_2_ and high humidity, Esco CelCulture^®®^ CO_2_ Incubator, CCL-170B-8-IVF, Esco Micro Pte. Ltd., Singapore). After 24h, the fibroblasts were treated with caffeine at a 5–1000 μM concentration range for evaluating the cytotoxicity. For the evaluation of the protective effect against oxidative stress, the cells were pretreated with free caffeine or the complex of caffeine and cyclodextrin in 5, 10, 50, 100, 500, and 1000 µM concentrations. Then, 500 µM H_2_O_2_ was applied for 1h, the cells were washed with PBS (with Ca^2+^ and Mg^2+^), and fresh medium was added. Neutral red solution (40 µg/mL) was added to each well (100 µL), and the plates were incubated for 3 h at 37 °C. After that, phosphate-buffered saline (PBS) was used for washing, and destaining solution (100 µL per well) was added. The plates were rapidly shaken for 10 min, and Synergy 2 multiplate reader (BioTek Instruments, Inc., Highland Park, Winooski, VT, USA) was used for the measurement of the optical density at 540 nm.

### 4.5. Incorporation of Caffeine Complex in Carbopol Hydrogel

**Figure 8:**
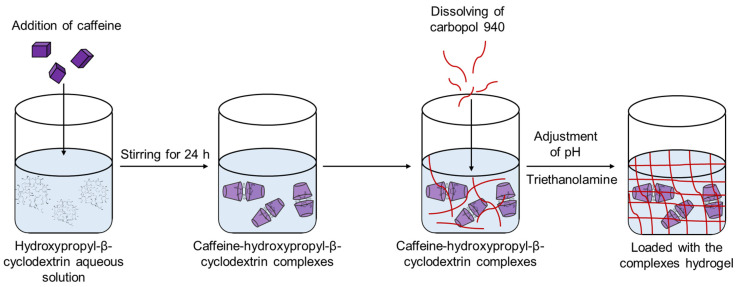
Schematic diagram of the preparation of carbopol hydrogel loaded with the caffeine–hydroxypropyl-β-cyclodextrin complex.

Carbopol 940 was used as a gelling agent for the preparation of the hydrogel (Figure 8). As illustrated, carbopol was added to the aqueous solution of the caffeine complex, and after its swelling, triethanolamine was added in order to increase the pH of the mixture. The sample was gently stirred until a hydrogel was formed. The concentration of carbopol in the hydrogel was 1% (wt/wt).

### 4.6. Characterization of the Hydrogel Formulation

The spreadability of the empty hydrogel and the hydrogel loaded with the complex was determined via the parallel-plate method [52,53]. In particular, the hydrogel (1 g) was placed between two glass plates (20 × 20 cm). Then, 250, 500, and 750 g weights were placed onto the upper glass subsequently for 3 min. The diameter of the sample between the plates (d) was measured. The spreadability and spreadability factors were determined according to the following equations:

S = d^2^ × π/4, (3)

where S represents the spreadability of the hydrogel sample (mm^2^) and d is the diameter (mm).

S_f_ = S/P, (4)

where S_f_ represents the spreadability factor of the hydrogel sample (mm^2^/g) and P is the charging weight (g).

The consistency of the hydrogels was determined via the pharmacopoeial penetrometry test. Briefly, after the preparation of the hydrogels, they were stored in the test container for 24 h at 25 ± 0.5 °C prior to testing. The gravity-driven penetrating object was released for 5 s, and the penetration depth was measured in millimeters.

A HAAKE MARS 60 rheometer with a parallel plate sensor system (top plate diameter = 20 mm; gap = 1.5 mm) was used for performing dynamic rheological measurements of the hydrogels. Three runs of each sample were conducted in controlled deformation mode. The oscillation amplitude sweep tests were carried out at 32 °C at a frequency of 1 Hz in the γ range from 0.001 to 20. Frequency sweep tests were conducted at 32 °C and constant deformation (γ = 0.002) in the 0.1–10 Hz frequency range.

### 4.7. In Vitro Dissolution Tests

In vitro dissolution tests were performed in citrate buffer (pH of 5.0) at 32 °C and gentle shaking (IKA Labortechnik HS-B20, Staufen, Germany). A total of 500 mg of the hydrogel, loaded with 15 mg caffeine in complex, was introduced into 50 mL of the medium. Then, 2 mL samples from the medium were taken at predetermined time intervals. The same volume of fresh medium was returned at each time point. In parallel, the same conditions were applied for the evaluation of the in vitro dissolution of pure caffeine. The concentration of the released caffeine was determined spectrophotometrically as described above.

### 4.8. Statistical Analysis

The experiments were conducted in triplicate, and the results are expressed as mean values ± SD. GraphPadPrism software, version 8 (GraphPad Software, San Diego, CA, USA), was applied for the statistical processing of the data. One-way ANOVA with Dunnett’s multiple comparison post-test and multiple *t*-tests, followed by the Holm–Sidak post-test, were performed.

## Figures and Tables

**Table 1:** Intrinsic solubility (S_0_), stability constant (K_1:1_), and complexation efficiency (CE) of the caffeine–hydroxypropyl-β-cyclodextrin complex.

S_0_, M	K_1:1_, M^−1^	CE
57.7	37.44	2.16

## Data Availability

The original contributions presented in this study are included in the article. Further inquiries can be directed to the corresponding authors.
